# Development and clinical evaluation of a rapid antibody lateral flow assay for the diagnosis of SARS-CoV-2 infection

**DOI:** 10.1186/s12879-021-06568-9

**Published:** 2021-08-23

**Authors:** Kesheng Li, Chongxiang Tong, Xiaoqin Ha, Chaoning Zeng, Xia Chen, Feifei Xu, Jinhong Yang, Huifen Du, Yuxin Chen, Jing Cai, Zengwei Yang, Zhongyi Jiang, Dandan Chai, Xueliang Zhang, Xun Li, Junfeng Li, Liqiong Yao

**Affiliations:** 1 Lanzhou Yahua Biotechnology Ltd. Co, Lanzhou, China; 2 Clinical Lab, Lanzhou Lung Hospital, Lanzhou, China; 3 Clinical Lab, 940 Hospital of Joint Logistic Support, People’s Liberation Army, Lanzhou, China; 4 Clinical Lab, Nanjing Gulou Hospital, Nanjing, China; 5 Pathogeny Biology Lab, Gansu Disease Control and Prevention Center, Lanzhou, China; 6 Department of Medicine Biotechnology, Gansu Provincial Academic Institute for Medical Research, Lanzhou, China; 7 Institute of Infectious Disease, The 1st Hospital, Lanzhou University, Lanzhou, China

**Keywords:** Lateral flow assay, SARS-CoV-2 antibody test, SARS-CoV-2 diagnosis, Clinical application value

## Abstract

**Background:**

The novel coronavirus disease 2019 (COVID-19) is an infectious disease caused by severe acute respiratory syndrome coronavirus 2 (SARS-CoV-2), which has quickly spread worldwide since its outbreak in December 2019. One of the primary measures for controlling the spread of SARS-CoV-2 infection is an accurate assay for its diagnosis. SARS-CoV-2 real-time PCR kits suffer from some limitations, including false-negative results in the clinic. Therefore, there is an urgent need for the development of a rapid antibody test kit for COVID-19 diagnosis.

**Methods:**

The nuclear capsid protein (N) and spike protein 1 (S1) fragments of SARS-CoV-2 were expressed in *Escherichia coli*, and rapid antibody-based tests for the diagnosis of SARS-CoV-2 infection were developed. To evaluate their clinical applications, the serum from COVID-19 patients, suspected COVID-19 patients, recovering COVID-19 patients, patients with general fever or pulmonary infection, doctors and nurses who worked at the fever clinic, and health professionals was analyzed by the rapid antibody test kits. The serum from patients infected with *Mycoplasma pneumoniae* and patients with respiratory tract infection was further analyzed to test its cross-reactivity with other respiratory pathogens.

**Results:**

A 47 kDa N protein and 67 kDa S1 fragment of SARS-CoV-2 were successfully expressed, purified, and renatured. The rapid antibody test with recombinant N protein showed higher positive rate than the rapid IgM antibody test with recombinant S1 protein. Clinical evaluation showed that the rapid antibody test kit with recombinant N protein had 88.56 % analytical sensitivity and 97.42 % specificity for COVID-19 patients, 53.48 % positive rate for suspected COVID-19 patients, 57.14 % positive rate for recovering COVID-19 patients, and 0.5−0.8 % cross-reactivity with other respiratory pathogens. The analytical sensitivity of the kit did not significantly differ in COVID-19 patients with different disease courses (p < 0.01).

**Conclusions:**

The rapid antibody test kit with recombinant N protein has high specificity and analytical sensitivity, and can be used for the diagnosis of SARS-CoV-2 infection combined with RT-PCR.

**Supplementary Information:**

The online version contains supplementary material available at 10.1186/s12879-021-06568-9.

## Background

The novel coronavirus disease 2019 (COVID-19) has quickly spread around the world since its outbreak in December 2019. COVID-19 is caused by a novel coronavirus [1], which is similar to 2003 severe acute respiratory syndrome coronavirus 2 (SARS-CoV) and 2012 Middle East respiratory syndrome-CoV. The International Committee on Taxonomy of Viruses named the novel coronavirus SARS-CoV-2. COVID-19 has been declared a public health emergency of international concern. One of the primary measures for controlling the spread of SARS-CoV-2 infection is an accurate assay for its diagnosis. After the outbreak of COVID-19, COVID-19 RT-PCR kits were developed and used for the clinical diagnosis of SARS-CoV-2 infection [2]. Although RT-PCR has high specificity and sensitivity, the kits can only be used in certified laboratories, require expensive equipment and trained technicians, and take an average of 2−3 h to complete. In addition, false-negative test results are another disadvantage of using RT-PCR kits for COVID-19 diagnosis. Therefore, RT-PCR kits are not suitable for the rapid diagnosis of SARS-CoV-2 infection or the identification of asymptomatic infection. Confirmed COVID-19 cases have clinical symptoms in common, including fever, cough, myalgia or fatigue [3], and specific computed tomography imaging characteristics [4]. It has been reported that asymptomatic carriers can spread SARS-CoV-2 [5, 6]. Thus, screening asymptomatic carriers is also very important for controlling the spread of COVID-19. To this end, there is an urgent need to develop a rapid, simple, specific, sensitive, and accurate test for COVID-19 diagnosis.

SARS-CoV-2 antibodies in the serum of infected patients may be a reliable target for COVID-19 diagnosis. The body produces specific antibodies (IgM and IgG) during viral infection [7]. IgM antibody was detected in the serum of patients with SARS-CoV after infection for 3−6 days, and IgG antibody was detected after infection for 8 days [8, 9]. The SARS-CoV-2 antibody-producing process may be the same as that for SARS-CoV, because they belong to the same family of viruses and have a similar surface structure [10].

In this study, we expressed and purified the nuclear capsid protein (N) and spike protein 1 (S1) of SARS-CoV-2 in *Escherichia coli *(*E. coli*), and compared the immunoreactivity rates of recombinant N and S1 proteins in the COVID-19 serum. Next, we developed a rapid antibody test kit based on the lateral flow assay for COVID-19 diagnosis using the N protein, and evaluated the clinical application value of this rapid test kit for the diagnosis of SARS-CoV-2 infection. Our results showed that the rapid test kit with N protein had high specificity and sensitivity for the diagnosis of SARS-CoV-2 infection.

## Methods

### Study subjects and diagnosis

The study was approved by the Ethics Committee of the Pulmonary Hospital of Lanzhou (Lan fei lun: 2020-01). Serum samples were collected from 97 patients with COVID-19 and 41 patients with suspected COVID-19, as diagnosed by SARS-CoV-2 RT-PCR. Patients were treated at the Pulmonary Hospital of Lanzhou (Lanzhou, China) and Nanjing Gulou Hospital (Nanjing, China); 88 patients had general fever or pulmonary infection (COVID-19-negative) in the outpatient service of 940 Hospital of Chinese People’s Liberation Army Joint Logistic Support, 124 had *Mycoplasma pneumoniae* infection, 185 patients with respiratory tract infection were treated at the 940 Hospital, 48 doctors and nurses worked in the fever outpatient service of the 940 hospital (COVID-19-negative), and 56 health professionals underwent a medical examination in the health examination center of Gansu Province Tumor Hospital (Gansu Province, China).

### Establishment of recombinant plasmids encoding the N and S1 fragments of SARS-CoV-2

Full-length N and S1 proteins of SARS-CoV-2 were synthesized according to the published sequence (General Biosystems (Anhui) Co. Ltd., Anhui, China). The full-length N gene was ligated into the pET-28a vector, which was used to transform DH5*α E. coli* cells. Colonies were selected on Luria Bertani (LB) agar plates containing 30 µg/mL kanamycin. Recombinant plasmids were extracted using a Plasmid DNA Miniprep Kit (Qiagen, Hilden, Germany), and analyzed for the presence of the N gene by sequencing and the use of BamH I and Xho I restriction enzymes. The recombinant plasmid expressing the S1 gene was acquired and analyzed by the same procedure, except that the pET-30a vector and BamH I and Apa I restriction enzymes were used. To express the N protein and S1 protein, recombinant plasmids encoding the N protein (pET-28a-SARS-CoV-2-N) and S1 protein (pET-30a-SARS-CoV-2-S1) were transformed into *E. coli* BL21 (DE3) cells. Transformants were selected on kanamycin (30 µg/mL) plates. BL21 (DE3) cells containing recombinant plasmids were cultivated in 5 mL LB broth and the protein expression was induced by the addition of 1 mM IPTG, followed by shaking for 3 h at 37 °C. The bacterial pellet was subjected to sodium dodecyl sulfate polyacrylamide gel electrophoresis (SDS-PAGE) to analyze expression of the recombinant protein.

### SDS-PAGE analysis

To analyze expression of the recombinant N protein, 100 mL of the induced cultures were centrifuged and re-suspended in 10 mL sample buffer (10 mM phosphate-buffered saline (PBS), pH 7.4) and sonicated 60 times with 5-min pulses at 5-min intervals. The supernatant and pellet were separated by centrifugation at 10,000 rpm at 4 °C for 20 min. The pellet was washed once using 2 M urea and centrifuged. Finally, the pellet was dissolved in 8 M urea, after which the supernatant, 2 M urea solution, and 8 M urea solution were analyzed by SDS-PAGE. To analyze expression of the recombinant S1 protein, 100 mL culture was sonicated in buffer (50 mM Tris, pH 8.5) 100 times with 5-min pulses at 5-min intervals. The supernatant and pellet were separated by centrifugation at 10,000 rpm at 4 °C for 20 min, and the pellet was washed with 2 M urea and dissolved in 8 M urea as above. Finally, the supernatant, 2 M urea solution, and 8 M urea solution were analyzed by SDS-PAGE.

### Purification of recombinant N protein and S1 protein

To purify the N protein, after sonication, the supernatant was loaded onto the Ni-NTA column and eluted with 10 mM PBS (pH 7.4) containing 100 mM imidazole. The purified recombination N protein was analyzed by SDS-PAGE. To purify the S1 protein, the pellet was re-suspended in 10 mL of 2 M urea and centrifuged at 10,000 rpm at 4 °C for 20 min. The supernatant was discarded and the pellet was dissolved in 6 mL of 8 M urea. To obtain active proteins, the denatured recombination protein was re-folded in 50 mM Tris (pH 8.5) buffer containing 150 mM NaCl by decreasing the concentration of urea from 8 M to 3 M. The refolded protein was purified by gel filtration on the Sephacryl S-300 column (US Pharmacia International, Inc., Rockville, MD, USA) and analyzed by SDS-PAGE.

### Preparation of SARS-CoV-2 antibody lateral flow assay with purified recombinant N protein

**Fig. 1:**
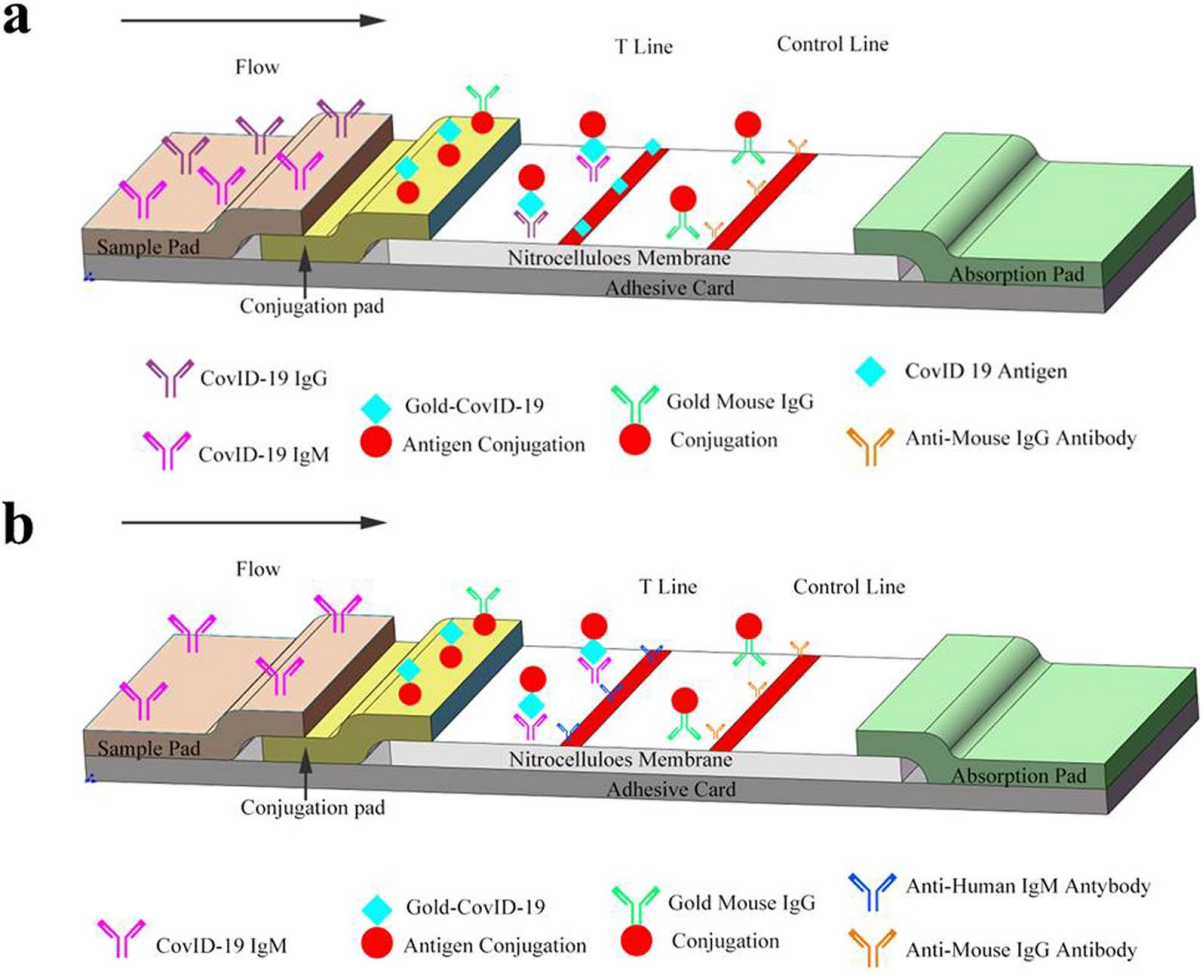
Overview of the SARS-CoV-2 antibody lateral flow assay. **a** SARS-CoV-2 antibody lateral flow assay with purified recombinant N protein. **b** SARS-CoV-2 antibody IgM lateral flow assay with purified recombinant S1 protein

**Fig. 2:**
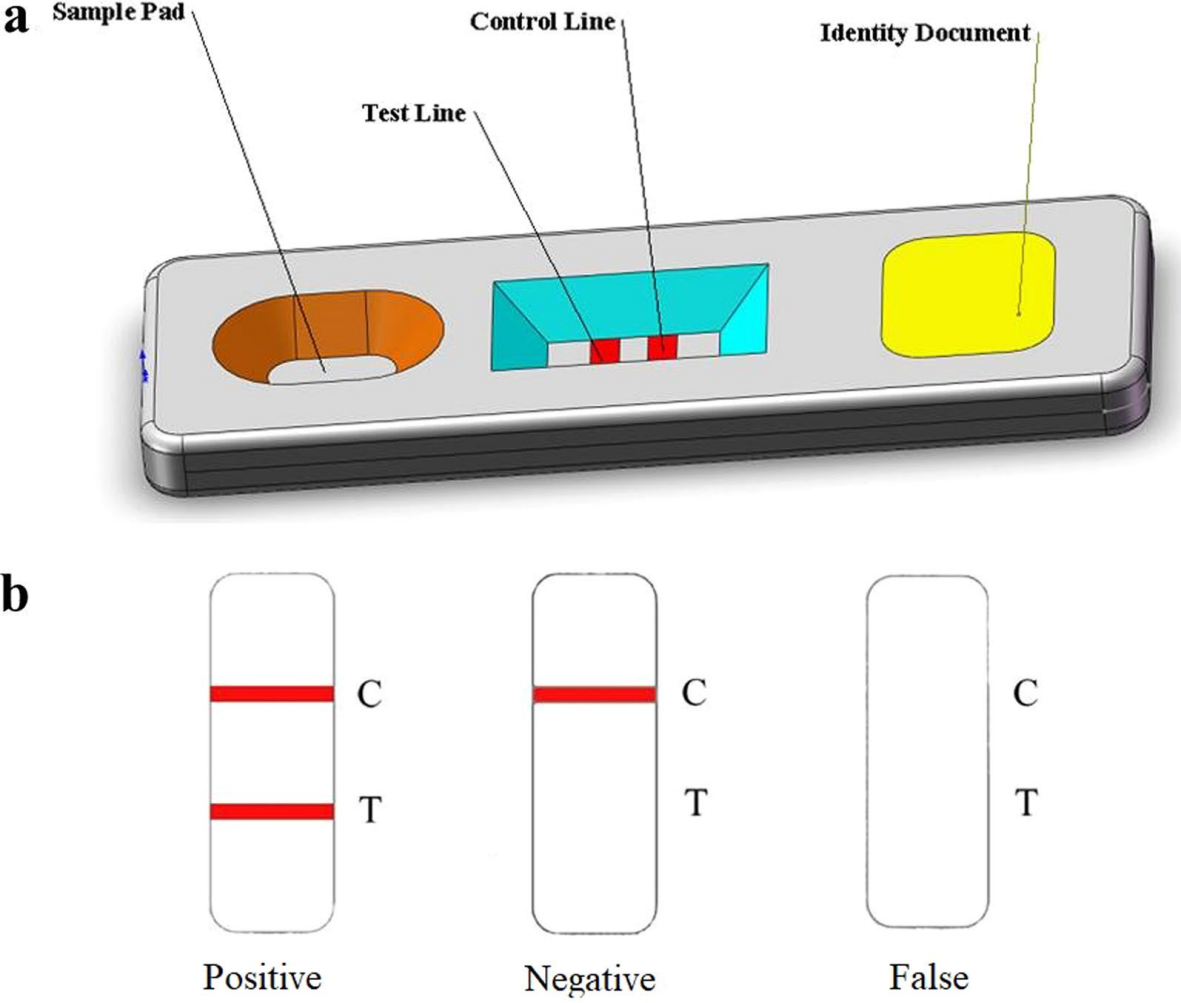
Structure of the special plastic box and exhibition of the testing result judgment. **a** Structure of the special plastic box. **b** Exhibition of the testing result judgment

The double-antigen sandwich colloidal gold method was used to prepare the SARS-CoV-2 antibody lateral flow assay with purified recombinant N protein. Specifically, the N protein was used to be immobilized at the test line (T line) and to prepare a recombinant N protein-colloidal gold conjugate at the same time. This assay can be used to detect the IgA, IgG and IgM antibody of the samples.To prepare a recombinant N protein-colloidal gold conjugate, 0.2 mg/mL, 0.4 mg/mL, 0.6 mg/mL, 0.8 mg/mL, 1.0 mg/mL, 1.2 mg/mL and 1.4 mg/mL purified recombinant N protein was respectively added to 100 mL of 0.1 % (w/v) colloidal gold (pH 7.5, 8.0, 8.3, 8.5, 8.8, 9.0, adjusted using 0.2 M potassium carbonate buffer), followed by 20 min of magnetic stirring and subsequent addition of 500 mg bovine serum albumin (BSA). After 10 min of magnetic stirring and 2 h incubation at 4 °C, the colloidal gold solution was centrifuged at 1000 rpm and 4 °C for 60 min. Then the supernatant was discarded, and the sediment (recombinant N protein-colloidal gold conjugate) was re-dissolved in 0.02 M (pH 7.4) Tri-HCI buffer containing 0.5 % BSA (w/v) and 0.1 % ProClin (w/v), and stored at 4 °C before use. The anti-human IgG monoclonal antibody (mAB)-colloidal gold conjugate was prepared using the same procedure as that used for the recombinant N protein-colloidal gold conjugate. The recombinant N protein-colloidal gold conjugate and anti-human IgG mAb-colloidal gold conjugate were mixed at a 1:1 ratio and sprayed on fiberglass (1 × 30 cm) by a special coating machine. The sprayed fiberglass was dried at 25 °C for more than 15 h and used as a conjugate pad. To prepare the reaction membrane, 1.2 mg/mL recombinant N protein and 1 mg/mL anti-mouse IgG (Shanghai JieNing Biotechnology Co. Ltd., Shanghai, China) were immobilized at the test line (T line) and control line (C line) on a nitrocellulose membrane (Vivid™ 170; Pall Life Sciences, Port Washington, NY, USA) using a special coating machine. The reaction membrane was solidified at 45 °C for 1 h. The lateral flow assay consisted of five parts: plastic backing, sample pad, conjugate pad, absorbent pad, and reaction membrane. The sample pad was 1.8 × 30 cm fiberglass (No. 8964; Hangzhou Hangan Biotechnology Co. Ltd., Zhejiang, China) pre-treated with 0.02 M (pH 7.4) containing 1 % BSA (w/v) and 0.5 % Tween-20 (v/v). The absorbent pad was 300 g general filter paper. The lateral flow assay is summarized in Fig. 1a. The individual assays (3 × 65 mm) were done in a special plastic box (Fig. 2a) and sealed in aluminum pouches with a silica desiccant pellet. The packages were stored at room temperature.

### Preparation of the SARS-CoV-2 IgM antibody lateral flow assay with purified recombinant S1 protein

The immunocapture method was used to prepare the SARS-CoV-2 IgM antibody lateral flow assay with purified recombinant S1 protein. Specifically, the S1 protein was used to prepare a recombinant S1 protein-colloidal gold conjugate, and the anti-human IgM was immobilized at the T line. This assay can be used to detect the IgM antibody of the samples. The recombinant S1 protein-colloidal gold conjugate was prepared using the same procedure as described above. The recombinant S1 protein−colloidal gold conjugate and anti-human IgG mAb−colloidal gold conjugate were also mixed at a 1:1 ratio and sprayed on a fiberglass (1 × 30 cm) to prepare the conjugate pad using the same procedure as above. The reaction membrane was prepared as above, except that 1 mg/mL anti-human IgM (Lanzhou Rujie Biotechnology Co. Ltd., Lanzhou, China) was immobilized at the T line. The structure and components of the SARS-CoV-2 IgM antibody lateral flow assay were the same as those for the SARS-CoV-2 antibody lateral flow assay (Fig. 1b).

### Sample testing

The test began immediately after the pouch was opened. Briefly, 10 L serum or plasma samples were pipetted into the sample port of the test card followed by the addition of two drops (80−100 µL) of dilution buffer (0.01 M PBS, pH 7.2). The test result was acquired in 20 min. The sample was positive when the C line and T line both appeared red, and was negative when only the C line was red. If the C line did not appear red, the test was invalid, and the sample was tested using another test card (Fig. 2b).

### Statistical analysis

The specificity and analytical sensitivity of the lateral flow assay were calculated according to the following formulas: specificity (%) = [true negative numbers/(true negative numbers + false positive numbers)] × 100 %: analytical sensitivity (%) = [true positive numbers/ (true positive numbers + false negative numbers)] × 100 %. Analysis was performed using SPSS 23.0, and the chi-square test was applied to compare two proportions. p < 0.05 was considered to be statistically significant.

## Results

### Cloning of the N gene and S1 fragment of SARS-CoV-2

**Fig. 3:**
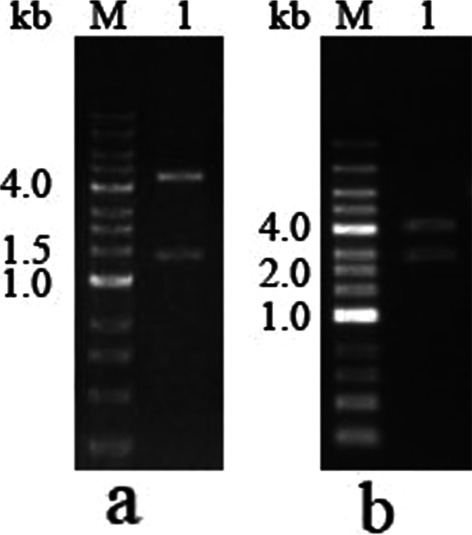
Digestion verification of the pET28a-SARS-2-N and pET30a-SARS-2-S1 plasmids. **a** Plasmid pET28a-SARS-CoV-2-N digested with BamH І and Xho І. M: DNA marker. 1: Plasmid pET28a-SARS-CoV-2-N. **b** Plasmid pET30a-SARS-CoV-2-S1 digested with BamH І and Apa І. M: DNA marker. 1: Plasmid pET30a-SARS-CoV-2-S1

The N gene (1257 bp) and S1 fragment (1800 bp) of SARS-CoV-2 were successfully cloned into pET28a and pET30a vectors, respectively. The sequence results of the cloned genes were similar to the published sequences [11]. The results of the digestion verification are shown in Fig. 3. The original image was shown in Additional file 1.

### Expression and purification of N protein and S1 protein of SARS-CoV-2

**Fig. 4:**
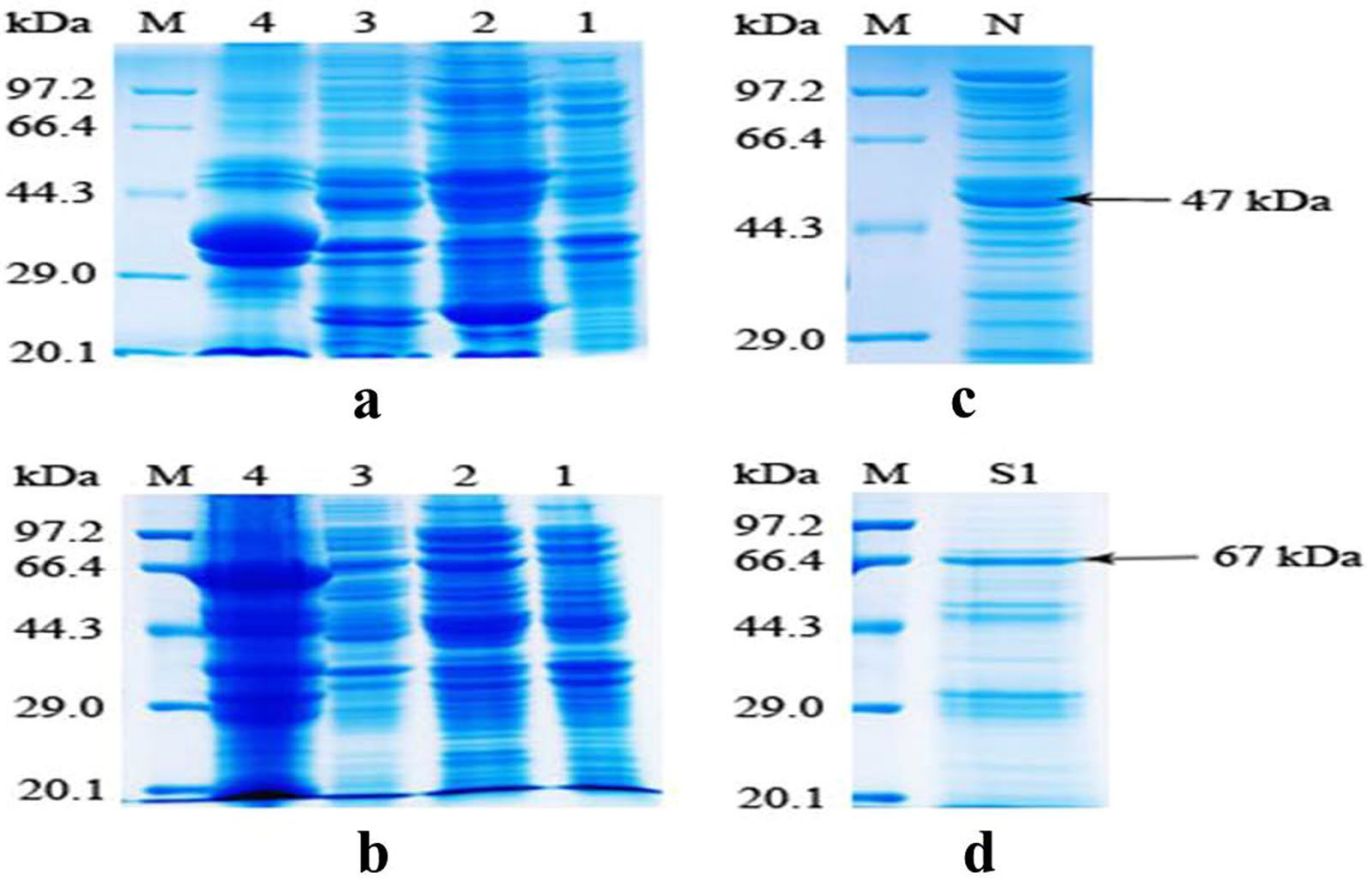
Expression and purification of N protein and S1 protein of SARS-CoV-2. **a** SDS-PAGE analysis of N protein showing its expression in *E. coli*. M: premixed protein marker. 1: protein extracts of uninduced *E. Coli*. 2: supernatant after sonication. 3: supernatant after washing pellets with 2 M urea. 4: 8 M urea solution of the pellet. **b** SDS-PAGE analysis of S1 protein showing its expression in *E. coli*. M: premixed protein marker. 1: protein extracts of uninduced *E. coli*. 2: supernatant after sonication. 3: supernatant after washing inclusion bodies with 2 M urea. 4: S1 protein dissolved in 8 M urea. **c** SDS-PAGE analysis of N protein after purification on the Ni-NTA column. M: premixed protein marker. N: N protein purified on the Ni-NTA column. **d** SDS-PAGE analysis of purified S1 protein. M: premixed protein marker. S1: purified S1 protein after re-folding

The N protein of SARS-CoV-2 was expressed in the soluble form (Fig. 4a). The N protein was purified by Ni-NTA column affinity chromatography, and a 47 kDa protein was obtained (Fig. 4c). The S1 protein of SARS-CoV-2 was expressed in inclusion bodies (Fig. 4b). The S1 recombinant protein was refolded as described in the Methods section, and a 67 kDa fragment was obtained (Fig. 4d). The original image was shown as Additional file 1.

### Positive analysis of the rapid antibody test kit with recombinant N protein and the IgM assay with recombinant S1 protein

The serum from 27 COVID-19 patients and 58 health professionals was tested simultaneously using the rapid antibody test kit with recombinant N protein and the IgM assay with recombinant S1 protein. The results showed that the positive rate of detection with the rapid antibody test kit with N protein was 92.59 % (25/27) for patients with COVID-19 and was 0.00 % (0/27) for health professionals; the positive rate of the IgM assay with S1 protein was 40.74 % (11/27) for patients with COVID-19 and 6.89 % (4/58) for health professionals (Table 1).

**Table 1:** Results of kit with recombinant N protein and IgM assay with recombinant S1 protein

Samples	Total samples (n)	Positive rate of antiboy rapid test kit with recombinant N protein (%)	Positive rate of IgM asasy with recombinant S1 protein (%)
Serum from patients with COVID-19	27	92.59 (25/27)	40.74 (11/27)
Serum from health persons	58	0.00 (0/58)	6.89 (4/58)

### Analytical sensitivity and specificity of the rapid antibody test kit with recombinant N protein

The serum from 97 COVID-19 patients, 58 health professionals, 88 patients with general fever or pulmonary infection, and 48 doctors and nurses who worked in the fever outpatient clinic were tested using the rapid antibody test kit with recombinant N protein. The results showed that 86 of 97 patients tested positive for COVID-19 (88.65 %), 0 of 58 health professionals (0.00 %), 4 of 88 patients with general fever or pulmonary infection (4.55 %), and 1 tested positive of the 48 doctors and nurses (2.08 %) (Table 2). The analytical sensitivity and specificity of the kit were 88.56 and 97.42 %, respectively.

### Comparative testing among COVID-19 patients, suspected COVID-19 patients, and those with general fever or pulmonary infection

The rapid antibody test kit was used to test the serum from 43 suspected COVID-19 patients, 97 COVID-19 patients, and 88 with general fever or pulmonary infection patients. The results showed that the suspected COVID-19 patients had a positive detection rate of 53.48 % (23/43), which was significantly higher than those with general fever or pulmonary infection (p < 0.01), and significantly lower than that in COVID-19 patients (p < 0.01; Table 3)

**Table 2:** Test results of the rapid antibody test kit with recombinant N protein

Samples	Total samples(n)	Positive rate (%)	p
Patients with COVID-19	97	88.65 (86/97)	0.000
Health persons	58	0.00 (0/58)	
General fever or pulmonary infection patients	88	4.55 (4/88)	
Doctors and nurses worked in fever outpatient service	48	2.08 (1/48)	

**Table 3:** Results of suspected versus confirmed COVID-19 patients and those with general fever or pulmonary infection

Samples	Total samples (n)	Positive rate (%)	p
COVID-19 patients	97	88.65 (86/97)	0.000
Suspected COVID-19 patients	43	53.48 (23/43)	0.000
General fever or pulmonary infection patients	88	4.55 (4/88)	

### Antibody testing at different times during the clinical progression of COVID-19

The serum obtained from 27 COVID-19 patients on days 1 and 10 after hospitalization and that obtained from recovering patients on day 14 after recovery were tested using the rapid antibody test kit. The results showed that the positive antibody detection rate of COVID-19 patients was 88.88 % (24/27) on day 1 and 92.59 % (25/27) on day 10, and that of recovering patients was 57.14 % (8/14) (Table 4). However, two patients had a negative antibody test during hospitalization, the antibody test results changed from positive to negative in two recovering patients, and one recovering patient maintained negative antibody results from infection initiation. Unfortunately, previous antibody test results were missing from three recovering patients who tested negative for COVID-19 with the rapid antibody test.

**Table 4:** Test results of antibody at different time points in COVID-19 patients

Time points	Total samples (n)	Positive rate (%)	p
First day	27	88.88 (24/27)	1.000
Tenth day	27	92.59 (25/27)	
Fourteenth day after recovery	14	57.14 (8/14)	

### Cross-reactivity

The serum obtained from patients infected with *M. Pneumoniae* and patients with respiratory tract infection were tested using the rapid antibody test. The results showed that there was 2 of 61 patients infected with *M. Pneumoniae* (3.27 %) and 4 of 125 patients with respiratory tract infection (3.20 %) had positive antibody results. Recently, we tried different methods to improve the specificity of the kit, and the samples was expanded. The results showed that only 1 of 124 patients infected with *M. Pneumoniae* (0.8 %) and 1 of 185 patients with respiratory tract infection (0.5 %) had positive antibody results. The rapid antibody test kit had 0.5−0.8 % cross-reactivity in patients infected with *M. Pneumoniae* and respiratory tract infection.

## Discussion

Laboratory investigations of serum antibody are important for the diagnosis of respiratory pathogen infection. Lateral flow assay based on the gold-based immunochromatographic assay is a very important tool to detect serum antibody, and has some advantages, including rapid testing, easy operation, and no need for an apparatus or site limitations. The lateral flow assay has been used for the diagnosis of many pathogens [12–15]. Because of the difficulty in obtaining natural antigen, recombinant protein has become the main diagnostic antigen for immunoassay for the diagnosis of pathogenic microorganism infection [11, 16, 17]. In this study, we successfully expressed N protein and S1 fragment of SARS-CoV-2 in *E. coli*, and developed a rapid antibody test kit and IgM assay for the diagnosis of SARS-CoV-2 infection using recombinant N protein and S1 fragment, respectively. The antibody test results from COVID-19 patients using the rapid antibody test with recombinant N protein had higher analytical sensitivity and specificity (88.56 % and 97.42 %) compared with the IgM assay with the recombinant S1 fragment (40.74 % and 93.11 %). There are several possible reasons for the lower sensitivity and specificity of the antibody IgM assay. First, the IgM assay only tested sera IgM antibody, and sera IgM levels in the majority of patients after infection are below the detection limit of the assay. However, the rapid antibody test with recombinant N protein could test total antibodies in the sera including IgA, IgM, and IgG. Second, the immune systems of patients have different antibody responses for various viral proteins (e.g., S, N, M, and E protein) after infection and have a stronger antibody response for N protein. Third, rheumatoid factors can cause false-positive results in the sera IgM assay, but the rapid antibody test kit does not have this issue because it is developed with a double-antigen sandwich. Therefore, the rapid antibody test kit with recombinant N protein is suitable for SARS-CoV-2 infection diagnosis. To evaluate the application of the rapid antibody test kit, 97 patients with COVID-19, 58 health professionals, 88 patients with general fever or pulmonary infection, and 48 doctors and nurses who worked in the fever outpatient clinic were analyzed, and the results demonstrated that it had 88.56 % sensitivity and 97.42 % specificity. Sensitivity of the rapid antibody test kit was in agreement with a recently reported kit, but its specificity was higher than that of the reported one, which is a rapid IgM-IgG combined antibody test kit developed using a recombinant receptor binding domain (RBD) of SARS-CoV-2 S protein with 88.66 % sensitivity and 90.3 % specificity [18]. The specificity difference of the two kits may be due to their antigens and preparation technology, because the rapid antibody test kit was developed using recombinant N protein and prepared by double-antigen sandwich, and the reported IgM-IgG combined antibody test kit was developed using the recombinant RBD of the S protein and prepared by the immunocapture method.

For clinical evaluation of the rapid antibody test kit in suspected COVID-19 patients, 43 suspected COVID-19 patients were analyzed. The test results showed 53.48 % positivity, which was significantly higher than the negative control (4.55 %), although significantly lower than that of COVID-19 patients (88.56 %). These results suggest that due to false-negative RT-PCR results, more than 50 % of suspected COVID-19 patients may have SARS-CoV-2 infection, and the possibility of positive cases of general fever or pulmonary infection in patients with COVID-19 cannot be ruled out. To clinically evaluate the rapid antibody test kit for COVID-19 patients at different points during disease progression, 27 COVID-19 patients at different points in their disease and 15 recovering patients were analyzed. The results demonstrated that the rapid antibody test kit had 88.88 % sensitivity on day 1 and 92.59 % sensitivity on day 10 after hospitalization in COVID-19 patients, and 57.14 % sensitivity on day 14 after recovery for recovering patients. Because the latent period of SARS-CoV-2 infection is 2−7 days, and the median is 4 days [3], the number of COVID-19 patients who produced specific antibodies on day 10 was more than that on day 1 after hospitalization. Therefore, the positive antibody detection rate in the preliminary stage of COVID-19 was lower than that in the later stage. These results demonstrated that a few COVID-19 patients could not produce specific antibodies, and the antibody levels of some recovering patients gradually decreased until they disappeared. To determine if there was any cross-reactivity between the rapid antibody test kit with other pathogenic microorganism infections, cross-reactivity experiments were conducted. The results demonstrated that the rapid antibody test kit had low cross-reactivity (0.5−0.8 %) in patients infected with the *M. Pneumoniae* respiratory pathogen. The reasons for the cross-reactivity are unclear and need further study.

There are some limitations in our research. First, the protein preparation and clearly the purity is significantly different between NiNTA of N protein and urea preparation of S1 proteins, which may affect sensitivity between peptides. Second, although immunological detection methods developed to detect virus-specific antibodies are mainly aimed at the structural proteins including S protein and N protein, it is well established that the most significant neutralization activity of antibodies produced by recovered patients is mediated by epitopes to S1 protein and the RBD domain, not N protein. Therefore, utility of N protein antibody tests will be limited in interpretation of recovering patients and of no use to vaccinated individuals.

## Conclusions

COVID-19 is a novel infectious disease. Diagnostic technology and assays are very important for controlling its spread. This study described a simple approach for producing full-length N protein of SARS-CoV-2 and developing a rapid antibody test kit for the diagnosis of SARS-CoV-2 infection. Clinical evaluation of the test showed that it had high analytical sensitivity and specificity, and low cross-reactivity with other respiratory pathogens. Thus, this rapid antibody test is a simple, rapid, and suitable assay for diagnosing SARS-CoV-2 infection in combination with RT-PCR.

## Supplementary information

**Additional file 1: Fig. S3. **Digestionverification of the pET28a-SARS-2-N and pET30a-SARS-2-S1 plasmids.** a** Plasmid pET28a-SARS-CoV-2-N digestedwith BamH І and Xho І. M: DNA marker. 1: Plasmid pET28a-SARS-CoV-2-N. **b** Plasmid pET30a-SARS-CoV-2-S1 digestedwith BamH І and Apa І M: DNA marker. 1: Plasmid pET30a-SARS-CoV-2-S1. **Fig. S4 **Expression and purification of Nprotein and S1 protein of SARS-CoV-2.** a**SDS-PAGE analysis of N protein showing its expression in *E. coli*. M: premixed protein marker. 1: protein extracts of uninduced*E. Coli*. 2: supernatant aftersonication. 3: supernatant after washing pellets with 2 M urea. 4: 8 M ureasolution of the pellet. **b** SDS-PAGEanalysis of S1 protein showing its expression in *E. coli*. M: premixed protein marker. 1: protein extracts ofuninduced *E. coli*. 2: supernatantafter sonication. 3: supernatant after washing inclusion bodies with 2 M urea.4: S1 protein dissolved in 8 M urea. **c**SDS-PAGE analysis of N protein after purification on the Ni-NTA column. M:premixed protein marker. N: N protein purified on the Ni-NTA column. **d** SDS-PAGE analysis of purified S1protein. M: premixed protein marker. S1: purified S1 protein after re-folding.

## Data Availability

The datasets used and analyzed during the current study are available from the corresponding author on reasonable request.

## Abbreviations

COVID-19: Coronavirus disease 2019
SARS-CoV-2: Severe acute respiratory syndrome coronavirus 2
N: Nuclear capsid protein
S1: Spike protein 1
RT-PCR: Reverse transcription-PCR
PBS: Phosphate-buffered saline
IPTG: Isopropyl β-D-thiogalactoside
SDS-PAGE: Sodium dodecyl sulfate polyacrylamide gel electrophoresis
BSA: Bovine serum albumin
mAB: Monoclonal antibody
T line: Test line
C line: Control line
RBD: Recombinant receptor binding domain
