# Supplementary material for: Development and clinical evaluation of a rapid antibody lateral flow assay for the diagnosis of SARS-CoV-2 infection
Source: BMC Infect Dis. 2021 Aug 23;21:860. doi: 10.1186/s12879-021-06568-9 (PMC8381135; doi:10.1186/s12879-021-06568-9)
Supplement: Supplementary file 1 — Additional file 1: Fig. S3. Digestionverification of the pET28a-SARS-2-N and pET30a-SARS-2-S1 plasmids. a Plasmid pET28a-SARS-CoV-2-N digestedwith BamH І and Xho І. M: DNA marker. 1: Plasmid pET28a-SARS-CoV-2-N. b Plasmid pET30a-SARS-CoV-2-S1 digestedwith BamH І and Apa І M: DNA marker. 1: Plasmid pET30a-SARS-CoV-2-S1. Fig. S4 Expression and purification of Nprotein and S1 protein of SARS-CoV-2. aSDS-PAGE analysis of N protein showing its expression in E. coli. M: premixed protein marker. 1: protein extracts of uninducedE. Coli. 2: supernatant aftersonication. 3: supernatant after washing pellets with 2 M urea. 4: 8 M ureasolution of the pellet. b SDS-PAGEanalysis of S1 protein showing its expression in E. coli. M: premixed protein marker. 1: protein extracts ofuninduced E. coli. 2: supernatantafter sonication. 3: supernatant after washing inclusion bodies with 2 M urea.4: S1 protein dissolved in 8 M urea. cSDS-PAGE analysis of N protein after purification on the Ni-NTA column. M:premixed protein marker. N: N protein purified on the Ni-NTA column. d SDS-PAGE analysis of purified S1protein. M: premixed protein marker. S1: purified S1 protein after re-folding. [file 12879_2021_6568_MOESM1_ESM.docx]

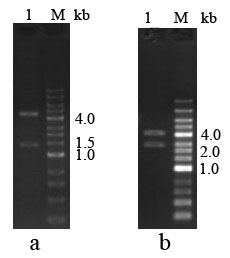


**Fig. 3** Digestion verification of the pET28a-SARS-2-N and pET30a-SARS-2-S1 plasmids. **a** Plasmid pET28a-SARS-CoV-2-N digested with BamH І and Xho І. M: DNA marker. 1: Plasmid pET28a-SARS-CoV-2-N. **b** Plasmid pET30a-SARS-CoV-2-S1 digested with BamH І and Apa І M: DNA marker. 1: Plasmid pET30a-SARS-CoV-2-S1.


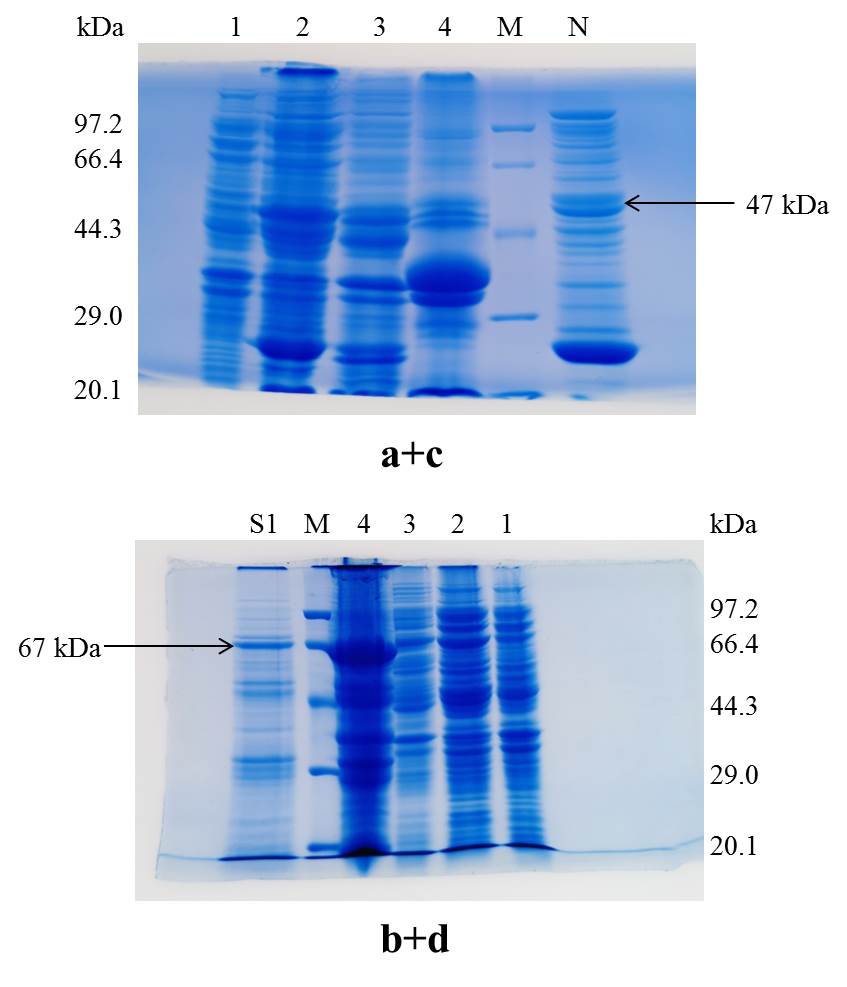


**Fig. 4** Expression and purification of N protein and S1 protein of SARS-CoV-2. **a** SDS-PAGE analysis of N protein showing its expression in *E. coli*. M: premixed protein marker. 1: protein extracts of uninduced *E. Coli*. 2: supernatant after sonication. 3: supernatant after washing pellets with 2 M urea. 4: 8 M urea solution of the pellet. **b** SDS-PAGE analysis of S1 protein showing its expression in *E. coli*. M: premixed protein marker. 1: protein extracts of uninduced *E. coli*. 2: supernatant after sonication. 3: supernatant after washing inclusion bodies with 2 M urea. 4: S1 protein dissolved in 8 M urea. **c** SDS-PAGE analysis of N protein after purification on the Ni-NTA column. M: premixed protein marker. N: N protein purified on the Ni-NTA column. **d** SDS-PAGE analysis of purified S1 protein. M: premixed protein marker. S1: purified S1 protein after re-folding.
